# Modeling and Comparing Brain Processes in Message and Earned Source Credibility Evaluation

**DOI:** 10.3389/fnhum.2022.808382

**Published:** 2022-05-06

**Authors:** Piotr Schneider, Grzegorz M. Wójcik, Andrzej Kawiak, Lukasz Kwasniewicz, Adam Wierzbicki

**Affiliations:** ^1^Department of Neuroinformatics and Biomedical Engineering, Institute of Computer Science, Maria Curie-Sklodowska University, Lublin, Poland; ^2^Polish-Japanese Academy of Information Technology, Warsaw, Poland

**Keywords:** credibility, EEG, sLORETA, trust and distrust, source localization

## Abstract

Understanding how humans evaluate credibility is an important scientific question in the era of fake news. Source credibility is among the most important aspects of credibility evaluations. One of the most direct ways to understand source credibility is to use measurements of brain activity of humans who make credibility evaluations. This article reports the results of an experiment during which we have measured brain activity during credibility evaluation using EEG. In the experiment, participants had to learn source credibility of fictitious students based on a preparatory stage, during which they evaluated message credibility with perfect knowledge. The experiment allowed for identification of brain areas that were active when a participant made positive or negative source credibility evaluations. Based on experimental data, we modeled and predicted human source credibility evaluations using EEG brain activity measurements with F1 score exceeding 0.7 (using 10-fold cross-validation). We are also able to model and predict message credibility evaluations with perfect knowledge, and to compare both models obtained from a single experiment.

## 1. Introduction

In the wake of the pandemic, we live in an information society that is struggling with a new social problem: global and fast spread of disinformation on the Web. While propaganda, conspiracy theories and urban legends or gossip have most likely been around ever since humans have developed language and civilization, the problem of online disinformation is different. The Web enables fast and low-cost publishing of disinformation, while at the same time providing incentives for spreading it, due to the possibility of monetizing Web users attention through online advertising. Numerous recent examples have amply demonstrated the importance of the problem of spreading disinformation on the Web: from the role of fake news in the presidential campaign and presidency of Donald Trump, to the case of disinformation about COVID-19 and vaccines against the virus.

Efforts aimed at combating the spread of online disinformation are focused on two areas: detection and debunking of disinformation, and increasing the awareness and skills of information consumers in evaluating credibility (also referred to as media literacy). While many of these efforts are promising, they would all benefit from a better understanding of how humans evaluate the credibility of information. Research in this area has stared from psychology and media science (Hovland and Weiss, 1951) and has continued actively since then (Lazer et al., 2018; McIntyre, 2018). Today, research in the area of social psychology has identified many reasons of why humans make wrong credibility evaluations (Rutjens and Brandt, 2018; Forgas and Baumeister, 2019). Yet, research in social psychology is based on declarative information that is in itself subject to bias and cannot reveal real, but unconscious reasons for making credibility evaluation (Viviani and Pasi, 2017; Self and Roberts, 2019).

Very little is known about the underlying phenomena that occur in the brain during credibility evaluation. In neuroinformatics and neuroscience, most research related to credibility has focused on lie detection (Wang et al., 2016; Meijer and Verschuere, 2017), which is based on the investigation of the brain activity of the author, and not the receiver of the message. Previous research has attempted to study source credibility evaluation separately using EEG (Kawiak et al., 2020b). In this work, we set a different goal. We aim to compare the brain processes involved in source credibility and message credibility evaluation directly, in a single experiment. We hypothesize that these two types of credibility evaluation will involve different processes in the brain.

It is known that credibility evaluations are affected by multiple criteria and can be quite complex (as complex as the evaluated information) (Tseng and Fogg, 1999; Kakol et al., 2017; Wierzbicki, 2018). Credibility evaluations can be impacted by the evaluated information itself (the message) or by the message's source (Hovland and Weiss, 1951). The evaluation of message credibility is, in turn, influenced by the knowledge and experience of the evaluator, as well as by the design and presentation of the message. Research efforts aimed at understanding the brain processes involved in credibility evaluation would have to deal with this complexity by adequately controlling the involved criteria and variables.

Our goal requires a more elaborate experimental design. In realistic situations, message and source credibility is usually mixed. We need to create an experimental condition where message credibility can be studied in isolation from source credibility, and vice versa. Moreover, message and source credibility evaluation are affected by different variables. As mentioned above, message credibility evaluation depends on the knowledge of the evaluator about the information contained in the message. On the other hand, source credibility evaluation requires knowledge about the past messages sent by a certain source. In an experiment, both of these kinds of knowledge will need to be controlled and separated. This means that ideally, during the part of the experiment used to study message credibility, participants should have no knowledge about the source and complete knowledge about the message, and vice versa, during the study of source credibility, participants should have no knowledge about the message, but very good knowledge about the sources.

A better understanding of the brain processes involved in both source credibility and message credibility evaluation will bring us closer to the far-reaching goal of creating a diagnostic method for evaluation of credibility of Web content through the use of EEG. Such a method would be free of biases and i would be able to reveal the full impact of disinformation on human brain. While this goal is quite difficult to reach, we hope that the contributions made in this article bring it closer. We were able to create predictive models of both message and source credibility evaluations. The models are interpretable and reveal huge differences between brain processes involved in the two kinds of credibility evaluation. Moreover, our source credibility model strongly confirms and validates results obtained in previous research (Kawiak et al., 2020b), confirming that we are indeed increasing our understanding of how the human brain evaluates source credibility.

## 2. Related Work

### 2.1. Source, Message, Media Credibility

The concept of credibility, similarly to the concept of trust, is grounded both in science as well as in common sense. Credibility has been subject to research by scientists, especially in the field of psychology and media science. One of the earliest theoretical works on credibility dates back to the 1950s. This influential work of the psychologist Carl Hovland (Hovland and Weiss, 1951) introduced the distinction between ***source, message, and media credibility***. Out of these three, two are a good starting point for a top-down study of the complex concept of credibility: source credibility and message credibility. These two concepts are closely related to the natural-language (dictionary) definitions of the term “credibility.” In the English language dictionary (Oxford Advanced Learner's Dictionary), credibility is defined as “the quality that somebody/something has that makes people believe or trust them.” When this definition is applied to a person (“somebody”), it closely approximates source credibility— an essential concept in real-life, face-to-face communication. However, we should notice as well that the dictionary definition of credibility can also be applied to “something”—the message itself. And, in many online environments, message credibility must be evaluated without the knowledge about the source.

Information scientists have studied credibility evaluations with the goal of designing systems that could evaluate Web content credibility automatically or support human experts in making credibility evaluations (Wawer et al., 2014; Liu et al., 2015; Kakol et al., 2017; Oshikawa et al., 2018; Zhou and Zafarani, 2020; Ansar and Goswami, 2021). This research effort is supported by large corporations, such as Google and Twitter—who have started the Google Fact Check Explorer^1^ and Twitter Birdwatch^2^, respectively. Both of these services are based on human credibility evaluations - for instance, to quote the description of Twitter Birdwatch: “Birdwatch allows people to identify information in Tweets they believe is misleading.”

However, human credibility evaluations are often subjective, biased or otherwise unreliable (Kakol et al., 2013; Rafalak et al., 2014), making it necessary to search for new methods of credibility evaluation, such as the EEG-based methods proposed in this article. State of the art research on automatic fake news detection uses machine learning models trained on datasets that contain human credibility evaluations (D'Ulizia et al., 2021). The research described in this article can be seen as a step toward obtaining more objective and reliable training data for automatic fake news detection.

### 2.2. Message Credibility

A search for the term “message credibility” on Google Scholar returns over 1,000 results (for an overview of recent publications, especially on the subject of Web content credibility, see Wierzbicki, 2018). Researchers from the media sciences have attempted to create scale for declarative measurements of message credibility (Appelman and Sundar, 2016). The importance of message credibility on social media has been recognized in many studies (Wierzbicki, 2018), for example in the area of healthcare (Borah and Xiao, 2018).

As defined by Hovland, message credibility is the aspect of credibility that depends on the communicated message, not on its source or the communication medium. As such, message credibility depends on all information contained in the message itself. Consider a Web page that includes an article. The entire Web page is (in the information-theoretic sense) a message communicated to a receiver. Message credibility can depend on the article's textual content, on images or videos embedded in the article, on Web page design and style, or even on advertisements embedded in the Web page.

This simple example shows that message credibility can be affected by many factors, or features of the message. Even if we limit ourselves to just the textual content of the message, message credibility is affected by both the semantic content of the message (its “meaning”) and by the pragmatic content of the message (its style, persuasiveness, sentiment, etc.) This is especially important since message credibility is usually evaluated rapidly. The work of Tseng and Fogg (Tseng and Fogg, 1999) introduced the concepts of “surface credibility” and “earned credibility,” both of which can be applied to message credibility. Surface credibility is the result of a fast and superficial examination of the message. Earned credibility is the result of a slower and more deliberative reasoning about the message. The two concepts are similar to Kahneman's distinction about the fast, heuristic-based System I and the slower, deliberative System II (Kahneman, 2011). Surface credibility is message credibility based on System I reasoning, while earned credibility is message credibility based on System II reasoning. Research results (Wierzbicki, 2018) have established that most users evaluate Web page credibility quickly, in a matter of minutes (3 min are enough for most Web page credibility evaluations). These results are relevant for our experiment design. In order to begin to understand brain activity during message credibility evaluation, we shall limit message design to a single aspect that can be rapidly evaluated.

### 2.3. Reputed and Earned Source Credibility

Tseng and Fogg also introduced the concept of “reputed credibility.” When compared to earned credibility, reputed credibility is a notion that bases credibility evaluations on prior assumptions, rather than knowledge learned from direct, first-hand interactions. The distinction between the two concepts can be applied to source credibility. Reputed source credibility is the evaluation of a source based on prior assumptions: for example, if an article on the Web is written by a well-known doctor or scientists, we may assume that the article is credible. On the other hand, earned source credibility is based on first-hand, repeated interactions with a source. For example, if a person on social media frequently shares disinformation, we may decrease that person's source credibility evaluation.

The distinction between reputed and earned source credibility is useful to understand the scope of this article. Previous work (Kawiak et al., 2020b) investigated brain process involved in reputed source credibility evaluation. In an experiment participants were informed about sources' frequency of correctly answering a question. This information was given prior to the experiment, and was therefore equivalent to reputed source credibility. In our work, experiment participants learn about the correctness of sources first-hand, by evaluating their responses. Therefore, in this article we report on our investigation of brain processes involved in earned source credibility evaluations, and compare it to models of reputed source credibility evaluations obtained in Kawiak et al. (2020b). Moreover, we also investigated message credibility (under perfect knowledge), allowing to directly compare (in a single experiment) models of brain processes involved in earned source credibility and message credibility evaluation.

## 3. Experiment Design

The aim of the experiment was to observe participant's brain activity during a task that involved credibility evaluation. The experiment was divides into two consecutive stages P1 and P2.

### 3.1. Experiment Participants

Our experiment had 73 male, right-handed subjects aged 21 – 22 (avg. 21.3, s.d 0.458). For 40 participants, the recorded EEG signal (in both stages of the experiment) was adequate for future analysis and the signal-to-noise ratio (SNR) was at a satisfactory level, all tasks (screens) were completed and the number of recorded epochs was large enough to carry out source localization.

In order to perform the experiment, we obtained the approval of the Universtity's Bioethical Commission (MCSU Bioethical Comission permission 13.06.2019).

### 3.2. Credibility Evaluation Task

The participants who had no knowledge of Japanese (which was checked during the recruitment process by questionnaire), were told that they were looking at results of a real Japanese language test taken by other students. Participants were informed that they would be checking the responses of 3 other students: S1 - Bruno, S2 - Cesar, and S3 - John.

The aim of the first stage of experiment was to simulate a holistic credibility evaluation task with controlled knowledge. Participants were shown the questions and answers of S1, S2, or S3, along with a correct answer. The knowledge of participants was, therefore, complete, although their initial knowledge of the message topic (Japanese language) was nil.

**Figure 1:**
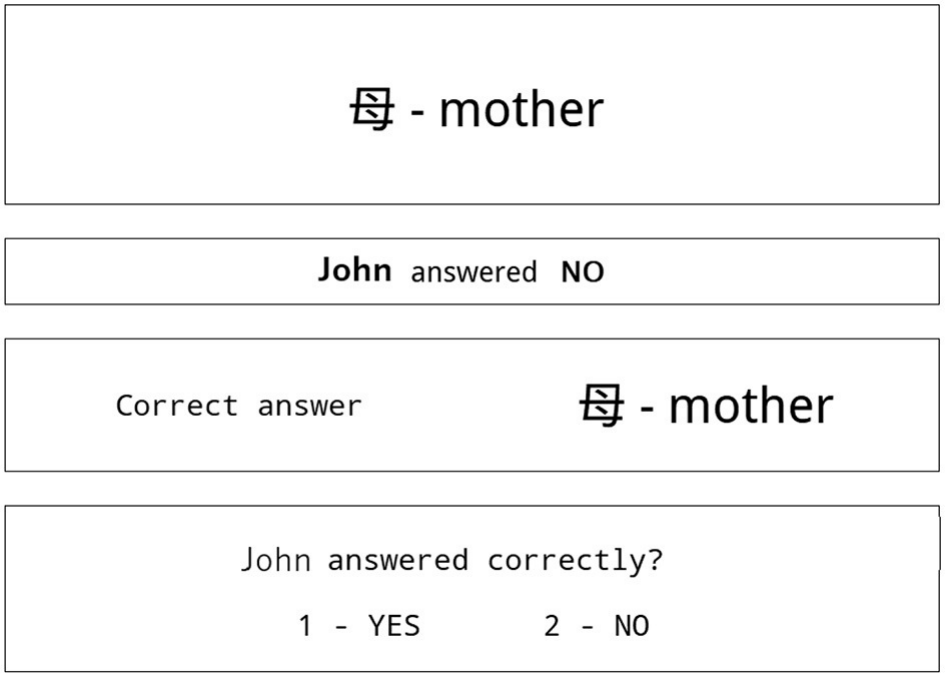
Typical screen shown to participant in stage 1 of the experiment.

A typical screen shown to participants in stage 1 of the experiment is shown in Figure 1. The screen is divided into 4 sections. All participants were informed about the screen design during an initial instruction before starting the experiment.

The first two sections of the screen originate from the test. The students taking the test were asked to evaluate the proposed meaning of some Kanji signs as true or false.

Consequently, the first section on top of the screen displays a Kanji sing and the intended question about the sign's meaning in the native language of participants.

The second section of the screen contains the responses of the student S1, S2, or S3. The response shows that according to the tested student, the meaning of the Kanji sign in the first section is true or false.

The following two sections are the hint and the task for our participants.

In the third section, there is a correct, dictionary meaning of the Kanji sign from Section 1.

The fourth section displays the task for the participant: to assess the response of the tested student as correct or incorrect.

During stage 1 of the experiment, participants were shown 408 screens: 136 for each student S1, S2, and S3. The first 136 screens consisted responses of S1, next 136 shown to participant consisted responses of S2, the last 136 screens consisted of student S3's responses on the fake test. Before the first screen of the assessment series of each student S1, S2, and S3, there was a screen informing participant that from now you he would be assessing the student: S1, S2, or S3 respectively.

The responses (shown to participants) of students S1, S2, and S3 were chosen in a special way:

- Student S1 was a weak student: he had only 25% of correct responses during the test.
- Student S2 was an average student: 50% of his responses were correct.
- Student S3 was quite a good student: 75% of his responses were correct.

Note that since in stage 1 of the experiment participants had perfect knowledge of the answer, but no previous knowledge of the students' performance, they were making their credibility evaluations based on message credibility. This design ensured a condition that after assessment of 136 responses of each student S1, S2, and S3, our participants knew which student is good, which one is poor and who is average. We checked this condition by asking participants about their opinion about the level of each student S1, S2, and S3 (using a special shown screen after stage 1 of the experiment).

**Figure 2:**
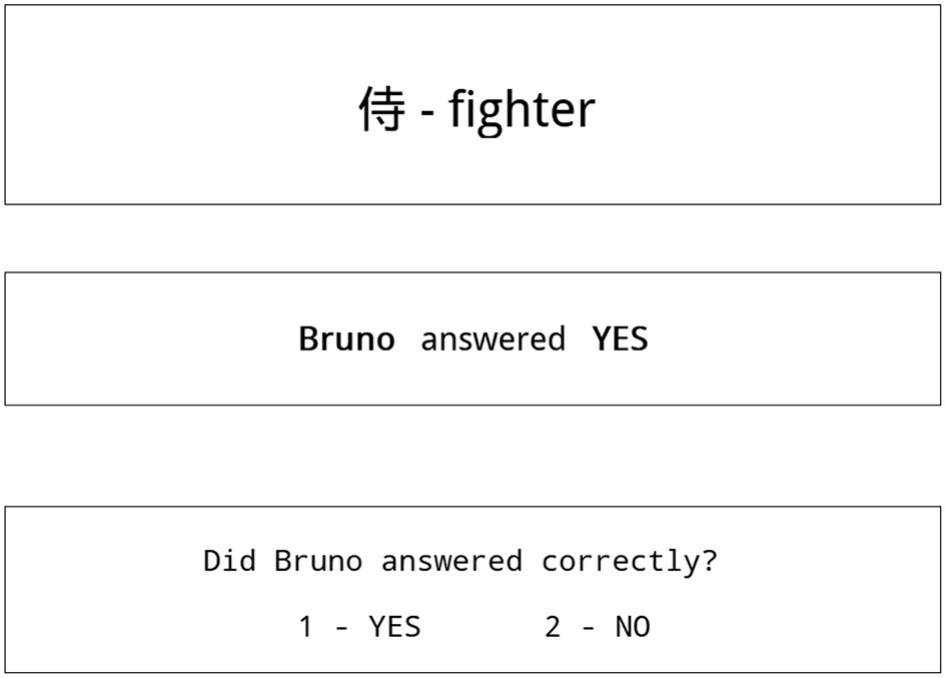
Typical screen shown to participant in stage 2 of the experiment.

Knowing the reputation of S1, S2, and S3, participants were asked to move on to stage 2. In stage 2 of the experiment, participants were shown screens similar to screens in the first stage, except there were no hints about the dictionary meaning of the Kanji sign (see Figure 2). Since participants now had no knowledge about the message, but knew the reputation of S1, S2, and S3, they were making their credibility evaluations based on message credibility.

Participants had to assess whether the student S1, S2, or S3 responded correctly basing only on their reputation learned in stage 1.

There were 102 screens in stage 2 of the experiment, 34 screens for each student S1, S2, and S3 but in contrast to stage 1—the screens of S1, S2, and S3 were presented to participants in random order.

### 3.3. Experimental Cases and Data

Based on the experiment setup, we were able to register EEG activity in 12 basic cases. These cases are similarly defined for stage 1 where participants had perfect knowledge (6 cases), and for stage 2 where participants had no knowledge (also 6 cases). The 12 experimental cases are defined in Table 1.

**Table 1:** The set of 12 Experiment Cases for 2 stages of the experiment.

**Experiment stage**	**Student accuracy**	**Evaluated message as**	**Evaluated message as**
		**Credible (C)**	**Not Credible (NC)**
Perfect knowledge	Weak student (S1)	$C_{S1}^{P1}$	$NC_{S1}^{P1}$
	Average student (S2)	$C_{S2}^{P1}$	$NC_{S2}^{P1}$
of message subject (P1)	Good student (S3)	$C_{S3}^{P1}$	$NC_{S3}^{P1}$
No knowledge	Weak student (S1)	$C_{S1}^{P2}$	$NC_{S1}^{P2}$
	Average student (S2)	$C_{S2}^{P2}$	$NC_{S2}^{P2}$
of message subject (P2)	Good student (S3)	$C_{S3}^{P2}$	$NC_{S3}^{P2}$

Note that during stage 1 of the experiment, participants had perfect knowledge, but had no knowledge of the student's performance. During stage 1, participants evaluated credibility based on message contents (message credibility). Knowledge of the student's accuracy in answering questions (student's reputation) was learned by the participants during stage 1 of the experiment.

However, our experiment design cannot fully guarantee that participants would not learn about student's accuracy faster than intended. This means that in the cases $C_{S1}^{P1}$, $NC_{S1}^{P1}$, $C_{S3}^{P1}$ and $NC_{S3}^{P1}$, participants may still be influenced by source credibility. For this reason, the most important cases in stage 1 of the experiment are $MC=C_{S2}^{P1}$ and $MNC=NC_{S2}^{P1}$. In these cases, participants evaluated student S2 who had an average accuracy of 50%, and, therefore, could not learn anything positive or negative about source credibility. For simplicity, we shall refer to these two special experimental cases during stage 1 as *MC* (Message Credible) and *MNC* (Message Not Credible).

The joint cases for stage 1 are: $C^{P1}=C_{S1}^{P1}\cup C_{S2}^{P1}\cup C_{S3}^{P1}$ and $NC^{P1}=NC_{S1}^{P1}\cup NC_{S2}^{P1}\cup NC_{S3}^{P1}$.

On the other hand, during stage 2 of the experiment, participants had no knowledge and had to rely on student's reputation. Therefore, they evaluated credibility based on source credibility. This experiment design allowed for comparing the two processes of message and source credibility evaluation.

However, the most clear effects in stage 2 should occur for the cases $C_{S1}^{P2}$, $NC_{S1}^{P2}$, $C_{S3}^{P2}$ and $NC_{S3}^{P2}$. This is because in these cases, participants can rely on the source credibility that they have learned in stage 1 about student S1 (accuracy of 25%) or student S3 (accuracy of 75%). For both students, there will be a strong impact of source credibility on the participant. This means that it will be interesting to compare two sums of cases: $SC=C_{S1}^{P2}\cup C_{S3}^{P2}$ and $SNC=NC_{S1}^{P2}\cup NC_{S3}^{P2}$. For simplicity, we shall refer to these two special experimental cases during stage 2 as *SC* (Source Credible) and *SNC* (Source Not Credible). For student S2, the impact of source credibility is expected to be weak even in stage 2 of the experiment.

The joint cases for stage 2 are: $C^{P2}=C_{S1}^{P2}\cup C_{S2}^{P2}\cup C_{S3}^{P2}$ and $NC^{P2}=NC_{S1}^{P2}\cup NC_{S2}^{P2}\cup NC_{S3}^{P2}$. We can also define joint cases that include all participants credibility evaluations (credible or not credible), but differ in the level of source credibility: $A_{S1}^{P2}=C_{S1}^{P2}\cup NC_{S1}^{P2}$, $A_{S2}^{P2}=C_{S2}^{P2}\cup NC_{S2}^{P2}$ and $A_{S3}^{P2}=C_{S3}^{P2}\cup NC_{S3}^{P2}$.

### 3.4. EEG Measurements

Our empirical experiments involved top EEG devices.

The laboratory is a complete and compatible system provided by electrical geodesic systems (EGI)^3^.

We were equipped with a dense array amplifier recording the cortical activity with up to 500 Hz frequency through 256 channels HydroCel GSN 130 Geodesic Sensor Nets provided by EGI. In addition, in the EEG Laboratory the Geodesic Photogrammetry System (GPS) was used, which makes a model of subject brain based on its calculated size, proportion, and shape, owing to 11 cameras placed in its corners, and then puts all computed activity results on this model with very good accuracy. The amplifier works with the Net Station 4.5.4 software, GPS under the control of the Net Local 1.00.00, and GeoSource 2.0. Gaze calibration, eye blinks, and saccades elimination are obtained, owing to the application of eye tracking system operated by SmartEye 5.9.7. The event-related potential (ERP) experiments are designed in the OpenSesame 3.2.8 environment.

The artifact removal system (eye-blinks, saccadic eye movements) were removed using the scripts implemented into the EGI system software which is not open source, however broadly described in Waveform Tools Technical Manual (EGI, 2006).

For the sLORETA source localization analyses (used for verification of the hypotheses) the ERP for all 256 electrodes had to be in fact calculated on the fly. However, one should note that research referred herein is not a typical ERP experiment. Having the ERP signal estimated for each one out of 256 electrodes, it was possible to calculate the mean electric charge (MEC) flowing through the BA situated under these electrodes on the brain cortex in so-called Cognitive Processing Time Range (CPTR). For the 1st stage of stage experiment the CPTR was between 420 and 520 ms, for the 2nd stage it was between 510 and 610 ms. The length of ranges was chosen by assessment of meaningful differences in shape for particular electrodes ERP plots. Thus, it was possible to conduct the full source localization analysis of the signal originating from all 256 electrodes using sLORETA algorithm (GeoSource parameters set as follow: Dipole Set: 2 mm Atlas Man, Dense: 2,447 dipoles Source Montages: BAs). Mean electric current flowing through each BA and varying in time was given as an output. Having those values calculated, it was possible to integrate that current in time and then get the MEC. The mean electric charge calculated for each electrode using source localization techniques could, as we intended, indicate the hyperactivity of some BAs that are not necessary precisely situated under the cognitive electrodes. For all calculations in both stages of experiment the CPTR was divided into 5 ms time intervals to calculate discrete MEC (dMEC) values. The idea and procedure of calculating MEC has been described in detail in Wojcik et al. (2018).

### 3.5. Experiment Hypotheses

Our experiment design allowed us to compare brain activity of message credibility evaluations and source credibility evaluations. We hypothesized that these two types of credibility evaluations involve different processes in the brain. This allowed us to formulate Hypothesis 1:

1. Cognitive ERP signals during source credibility evaluation and during message credibility evaluations have statistically significant differences (comparison of ERP signals based on cases: *C*^*P*1^ and *C*^*P*2^, *NC*^*P*1^ and *NC*^*P*2^).

Our experiment also allowed us to investigate the effect of source credibility on participants' decisions and cognitive ERP signals. Therefore, we formulated the following hypotheses:

2. Increase of source credibility increases the number of positive credibility evaluations made by participants (comparison of number of decisions in cases: $C_{S1}^{P2}$ and $NC_{S1}^{P2}$, $C_{S2}^{P2}$ and $NC_{S2}^{P2}$, $C_{S3}^{P2}$ and $NC_{S3}^{P2}$).

Hypothesis 2 concerned the existence of a relationship between the source credibility level (reputation of students' S1, S2, and S3), which was one of the main independent variables in our experiment, and the number of positive credibility evaluations. The validation of this hypothesis was a test of our experiment's internal validity.

Finally, we wished to investigate the ability of predicting participant's credibility evaluations based on MEC measurements in diverse Brodmann areas and time intervals. This led us to formulate the following hypotheses:

3. Participant's message credibility evaluations made in stage 1 of the experiment for student S2 can be predicted with high accuracy based on the mean electric charge flowing through various Brodmann areas in a certain time interval (cases: $MC=C_{S2}^{P1}$ and $MNC=NC_{S2}^{P1}$).
4. Participant's source credibility evaluations made in stage 2 of the experiment for students S1 or S3 can be predicted with high accuracy based on the mean electric charge flowing through various Brodmann areas in a certain time interval (cases: $SC=C_{S1}^{P2}\cup C_{S3}^{P2}$ and $SNC=NC_{S1}^{P2}\cup NC_{S3}^{P2}$).
5. The best model for classification of message credibility decisions based on cases: *MC* and *MNC* differs from the best model for classification of source credibility decisions (based on cases: *SC* and *SNC*).

Note that the validation of hypothesis 5 requires that the classifiers be interpretable, which excludes the use of black-box classifiers such as neural networks.

Finally, we wished to validate our results using the model obtained in Kawiak et al. (2020b). For this reason, we formulated the hypothesis:

6. The best model for classification of source credibility decisions (based on cases: $C_{S1}^{P2}\cup C_{S3}^{P2}$ and $NC_{S1}^{P2}\cup NC_{S3}^{P2}$) will be using as explanatory variables Brodman areas found significant for the classification of source credibility in Kawiak et al. (2020b).

## 4. Experiment Results

### 4.1. Efficacy of Learning in Part 1

In stage 1 of the experiment, participants were supposed to learn source credibility evaluations of the three fictitious students: S1, S2, and S3. After the first stage of the experiment was completed, participants were asked to assess students S1, S2, and S3. Our intention was to evaluate whether participants obtained full knowledge about the students' past performance. Only participants who had such knowledge were allowed to start the second stage P2. 30 out of 40 participants (for whom we had a signal of sufficient quality) were able to appropriately assess the knowledge level of fictitious students S1, S2, and S3.

The data was analyzed using **Python 3.8.5** and **scikit-learn 0.23.1**, **statsmodels 0.11.1** or **scipy 1.5.0** libraries.

### 4.2. Data Analysis

**Hypothesis 1 verification** Using the Mann-Whitney U test we have found statistically significant differences in all 88 Brodmann areas for both pairs of cases: *C*^*P*1^ and *C*^*P*2^, *NC*^*P*1^ and *NC*^*P*2^. The p-value for all 88 BAs is less then 0.001. The significance threshold was set to .05 Finding statistically significant differences in the signal for all BAs confirmed hypothesis 1.

**Figure 3:**
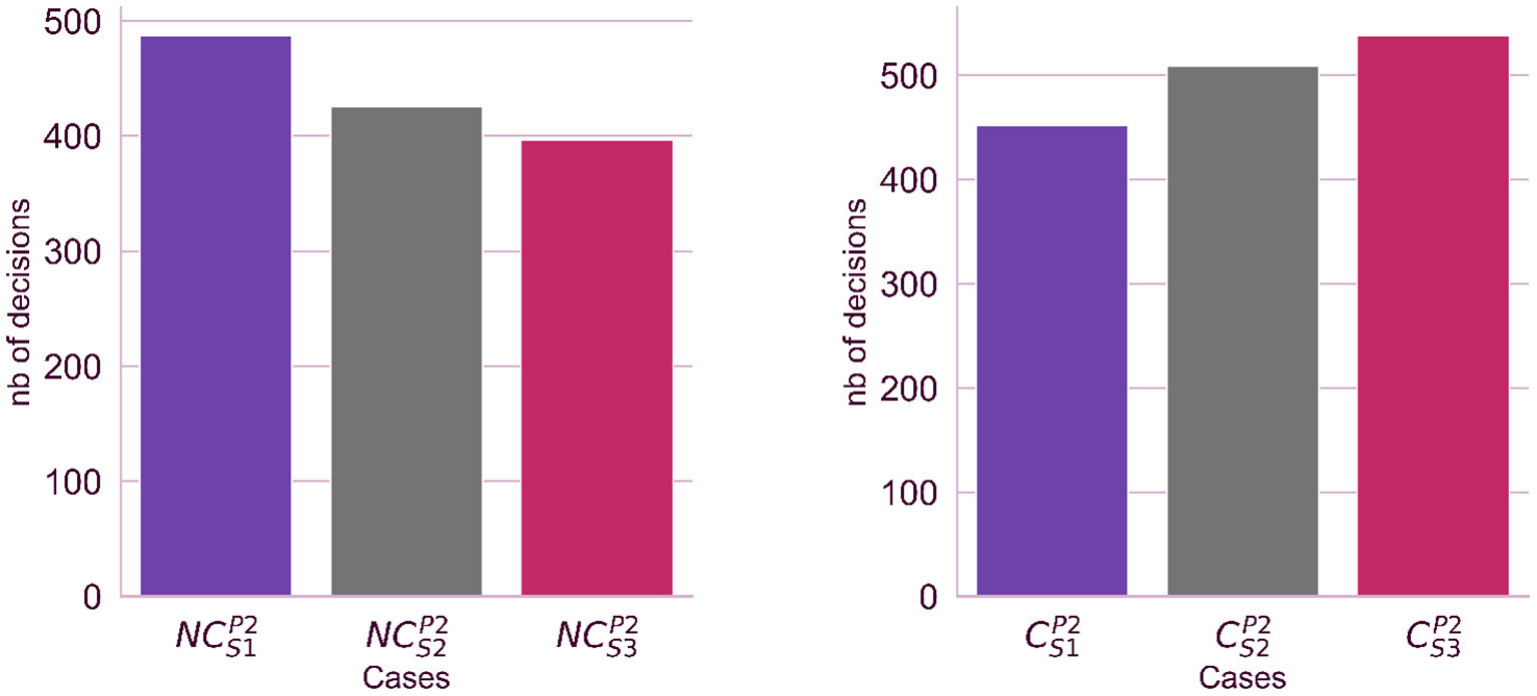
The impact of the source credibility on the numbers of decisions made by participants in the second stage of the experiment. We analyzed two cases: the participants considers the source as not credible (left side) or considers the source as credible (right side).

**Hypothesis 2 verification** Observing experiment logs and participant's responses we could confirm the existence of a relationship between the source credibility level (which increases respectively for students: S1-25%, S2-50%, and S3-75%) and the number of positive or negative credibility evaluations. The number of positive credibility evaluations is significantly larger when the student's source credibility is high (case $C_{S3}^{P2}$) and is smallest when the student's source credibility is low (case $C_{S1}^{P2}$). Similarly, the number of negative credibility evaluations is smallest for the student with high source credibility (case $C_{S3}^{P2}$) and increases as the student's source credibility rises (see Figure 3).

### 4.3. Data Preparation for Machine Learning Models

Before we started building a machine learning model, we had to select a machine learning algorithm and prepare data for the model.

Out of many machine learning algorithms, it was decided to use a logistic regression algorithm. It is characterized by simplicity in implementation and analysis of the obtained results, which was necessary for hypothesis verification. For both stages of the experiment, models that are binary classifiers were built, with the participant's credibility evaluation as the dependent variable.

Data preparation requires time interval selection, MEC standardization, dividing data into a training set and test set and limiting the number of independent variables for the model.

**Time interval selection** In order to select a time interval for MEC measurements, we examined the mean activity of BAs over time. We do that by creating line graphs for each participant. By analyzing the signal, we were able to determine the time period when the participant's brain was most active. Two intervals were selected by repeating the procedure for results of stage 1 of the experiment: 420–520 ms from stimulus, and for stage 2 of the experiment: 510–610 ms from stimulus.

**MEC Standarization**

In our previous research, we had used the mean electric charge (MEC) flowing through each BA in a given interval of time as independent variables of the machine learning models (Pascual-Leone et al., 2002; Liu et al., 2015; Wojcik et al., 2018; Kwaśniewicz et al., 2020). In this article we used a new approach. The MEC values are standardized using the StandardScaler method of the **scikit-learn 0.23.1** Python library. Standardization of a sample is a simple operation that uses the sample's mean and standard deviation. For each value in the sample, the standardized value is obtained by substracting the mean and dividing the result by the standard deviation. As a sample, we used the MEC measurements for a single BA in a given time period made for all participants. This means that the mean and standard deviation were obtained from MEC measurements of all participants. The brain cortex is covered by the mantle of meninges, the bones of the skull, the skin, the hair which results in a different SNR, and differing measurements of electrophysiological activity of various participants (Kwaśniewicz et al., 2021). That is why a standardization of the MEC is justified.

**Division of data into a training set and a test set** Thanks to the standardization of the dMEC signal we have obtained the continuum of signal for the whole cohort divided into 5 ms intervals. Such super-set of signal contains positive and negative credibility evaluations on all screens shown to all real participants of both stages of the experiment.

The data of the super-set was divided into the training and validation data sets. Eighty percent of the data was for used for training and 20% for validation. All transformations made on the data (e.g., standardization) were performed using transform pipelines. This method reduces data leakage in the machine learning process and no training set rows were repeated in the validation set.

**Limiting the number of independent variables for machine learning models**

In the beginning, we have dMEC values from 88 BAs. Using all the BAs we could probably build a model with good performance (accuracy), but difficult to interpret. Our goal was to build a model with good performance, but with as few independent variables as possible. To limit the amount of independent variables for the logistic regression model, the recursive feature elimination (RFE) method with 10-fold cross-validation with 3 replications was used. This method builds models with an increasing number of independent variables chosen from rankings of significance to the model. As a result of the method, for any number of independent variables (from 1 to the 88), we obtained 30 model accuracy (ACC) results. From these results, we calculated mean ACC and standard deviation. A box plot was created for each model. Analyzing box plots, we choose a model with a small number of independent variables and good performance. The particular set of independent variables (BAs) of the model was chosen automatically by the RFE method.

This process of eliminating independent variables was repeated separately for part 1 and part 2 of the experiment. For part 1, models have been built for cases: *C*^*P*1^ and *NC*^*P*1^. The process resulted in a choice of 32 independent variables corresponding to BAs from which the MEC was calculated in the chosen time interval (420–520 ms from stimulus). For part 2, the models have been built for cases: *C*^*P*2^ and *NC*^*P*2^. The process resulted in a choice of 26 independent variables corresponding to BAs from which the MEC was calculated in the chosen time interval (510–610 ms from stimulus).

The two sets of independent variables (32 for part 1, and 26 for part 2) were still quite large. For this reason, we repeated the RFE procedure for all models that were created to verify our hypotheses. We used the set of independent variables determined by the first application of the RFE procedure as an initial set, and applied the RFE procedure again, but with different classes for the models (for each the considered hypothesis, there are different experimental cases for classification). The results of the second application of the RFE procedure are described in the next section, separately for each hypothesis.

### 4.4. Machine Learning Models

**Hypothesis 3 verification**

In the first stage of the experiment, participants' credibility evaluations were divided into two main cases: MC and MNC. These cases consist of credibility evaluations made only for student S2 (see Section 3.3).

The purpose of building a machine learning model is to try to predict whether the participant's credibility evaluation belongs to the the case of MC or MNC. MC was treated as the positive class. The results of the model are presented in Table 2.

**Table 2:** Results obtained during data preparation process for the model to classify the cases of MC and MNC.

**Name**	**Value**
Time interval	420–520 ms
Independent variables (RFE algorithm)	32 BA
Independent variables (model)	6 BA
mean ACC	0.71
Standard deviation	0.035
Training data	1,134 observations
Test data	126 observations

The 32 BAs mentioned in Table 2 were obtained from the independent variable elimination process described in the previous section. We repeated this process for the model to predict cases MC and MNC. This time, we initially had 32 independent variables. Based on the box plots of the RFE procedure, we chose a model with only 6 independent variables: L-BA24, L-BA28, L-BA35, R-BA36, L-BA39, L-Hippocampus.

Finally, we built a classifier to predict cases MC and MNC using 6 independent variables. We used logistic regression estimator with 10-fold cross-validation with 3 replications. The estimator was with default values, but with the solver set to “newton-cg”. As a result, we obtained 30 ACC. From these results, we calculated mean ACC and standard deviation (see Table 2).

**Figure 4:**
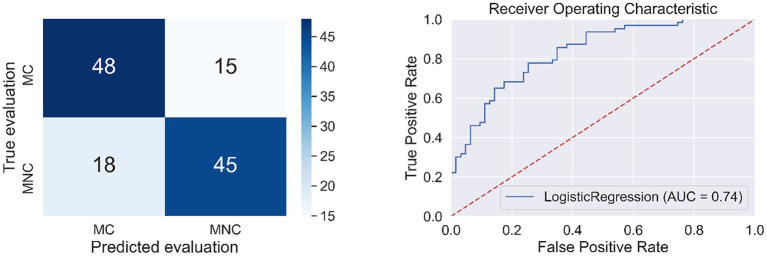
Confusion matrix (left side) and the ROC curve (right side) for the model to classify message credibility evaluations (the cases of MC and MNC).

The quality measures of the classifier are presented in Table 3 and Figure 4.

**Table 3:** Quality measures of the logistic regression model for predicting message credibility evaluations made in stage P1 of the experiment.

Accuracy	0.74
Precision	0.75
Recall	0.71
F1	0.73

**Figure 5:**
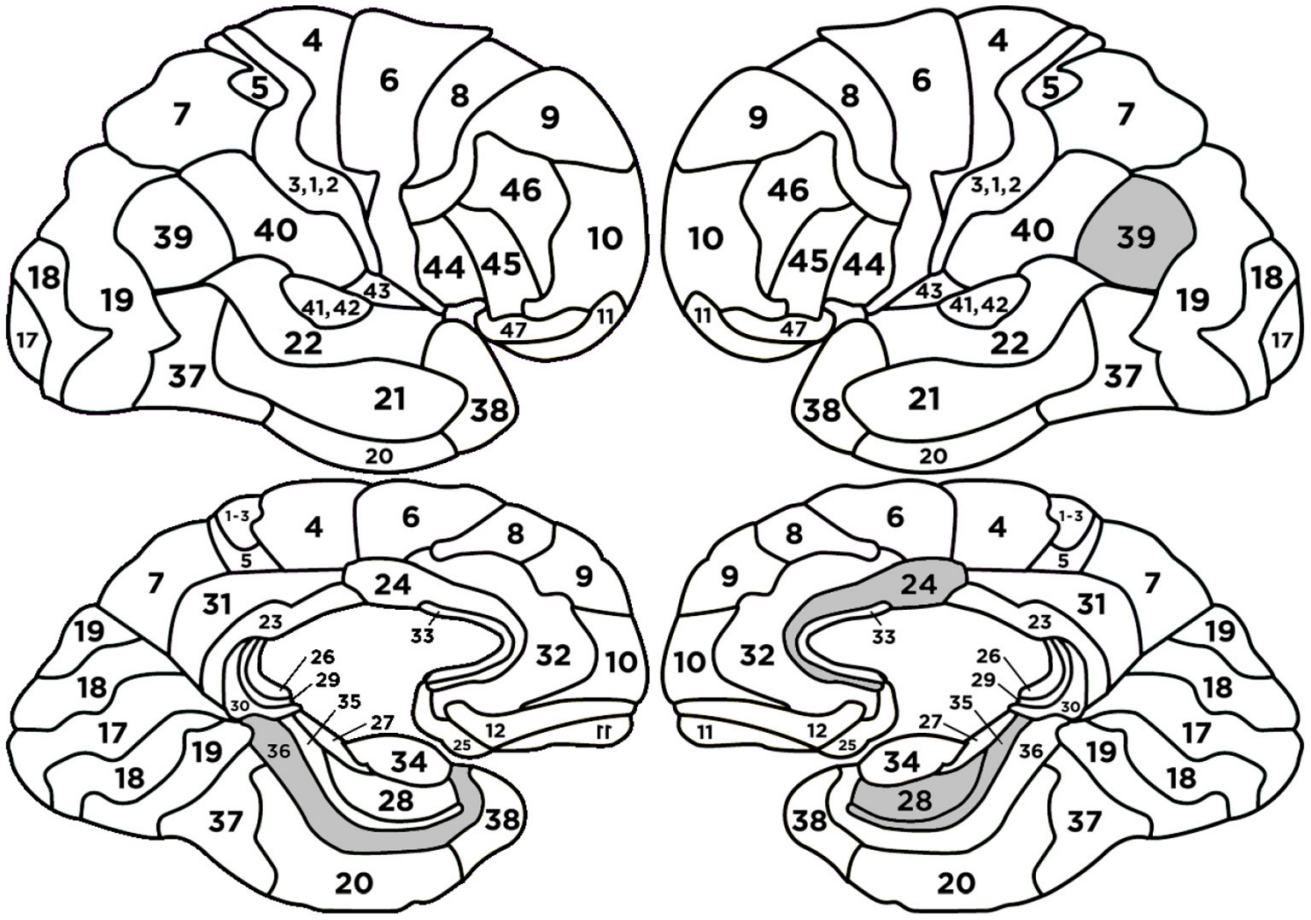
Heads with marked BAs that have the largest impact on the classification of message credibility evaluations (cases MC and MNC).

The following BAs: L-BA24, L-BA39, L-BA35 had positive regression parameters β equal to 1.831, 1.414, 1.184, respectively. The following BAs: R-BA36, L-Hippocampus, L-BA28 had negative regression parameters β equal to -2.735, -1.371, -1.297, respectively. The anatomical structures and their functionalities are presented in Appendix 4 and highlighted in Figure 5.

The presented results prove that we can build a good model for predicting message credibility, based on results of part 1 of our experiment.

**Hypothesis 4 verification**

In the second part of the experiment, participants' credibility evaluations were divided into two main cases: SC and SNC. These cases consist of credibility evaluations made for students S1 and S3 (see Section 3.3).

The purpose of building a machine learning model is to try to predict whether a participant's credibility evaluation belongs to the case of SC or SNC. SC was treated as the positive class. The results are presented in Table 4.

**Table 4:** Results obtained during data preparation process for the model to classify the cases of SC and SNC.

**Name**	**Value**
Time interval	510–610 ms
Independent variables (RFE algorithm)	26 BA
Independent variables (model)	10 BA
mean ACC	0.70
Standard deviation	0.027
Training data	2,268 observations
Test data	252 observations

The 26 BAs mentioned in Table 4 were obtained from the independent variable elimination process. We repeated this process for the model to predict cases SC and SNC. This time we initially had 26 independent variables. Based on the box plots of the RFE procedure, we chose a model with 10 independent variables: L-BA07, L-BA11, L-BA19, L-BA21, R-BA23, L-BA24, R-BA28, R-BA42, R-BA43, L-BA47.

Finally, we built a classifier to predict cases SC and SNC using 10 independent variables. We used logistic regression estimator with 10-fold cross-validation with 3 replications. The estimator was with default values, but with the solver set to “newton-cg”. As a result, we obtained 30 ACC. From these results, we calculated mean ACC and standard deviation (see Table 4).

**Figure 6:**
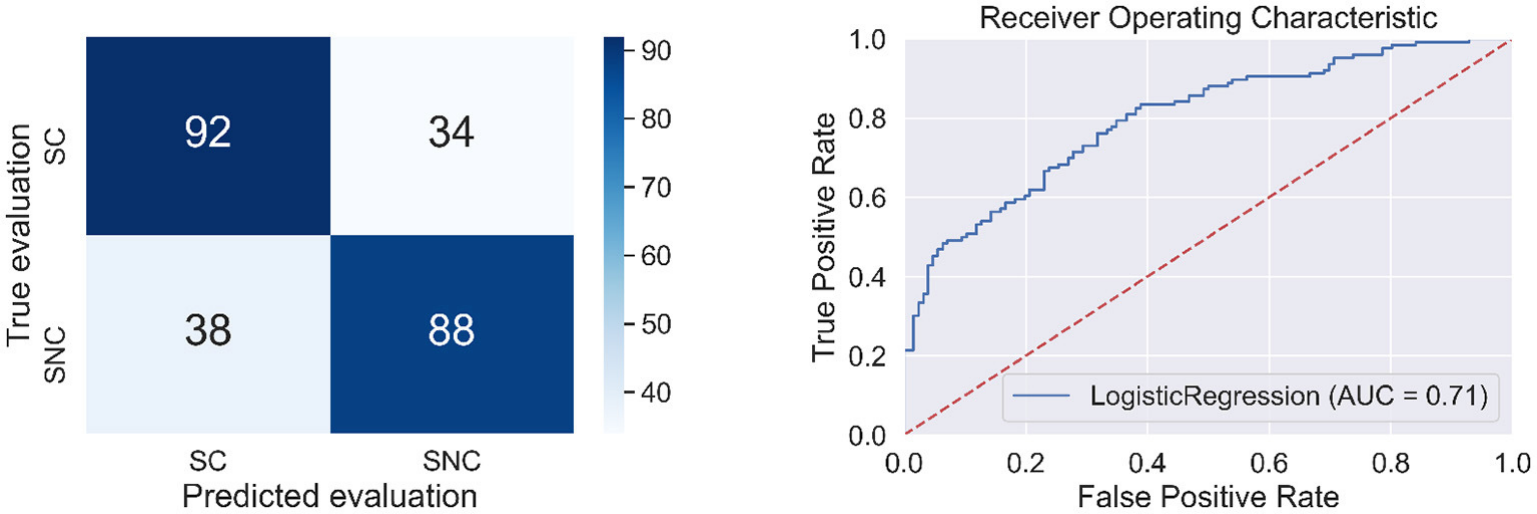
Confusion matrix (left side) and the ROC curve (right side) for the model to classify source credibility evaluations (the cases of SC and SNC).

The quality measures of the classifier are presented in Table 5 and Figure 6.

**Table 5:** Quality measures of the logistic regression model for predicting source credibility evaluations made in part 2 of the experiment.

Accuracy	0.71
Precision	0.72
Recall	0.70
F1	0.71

**Figure 7:**
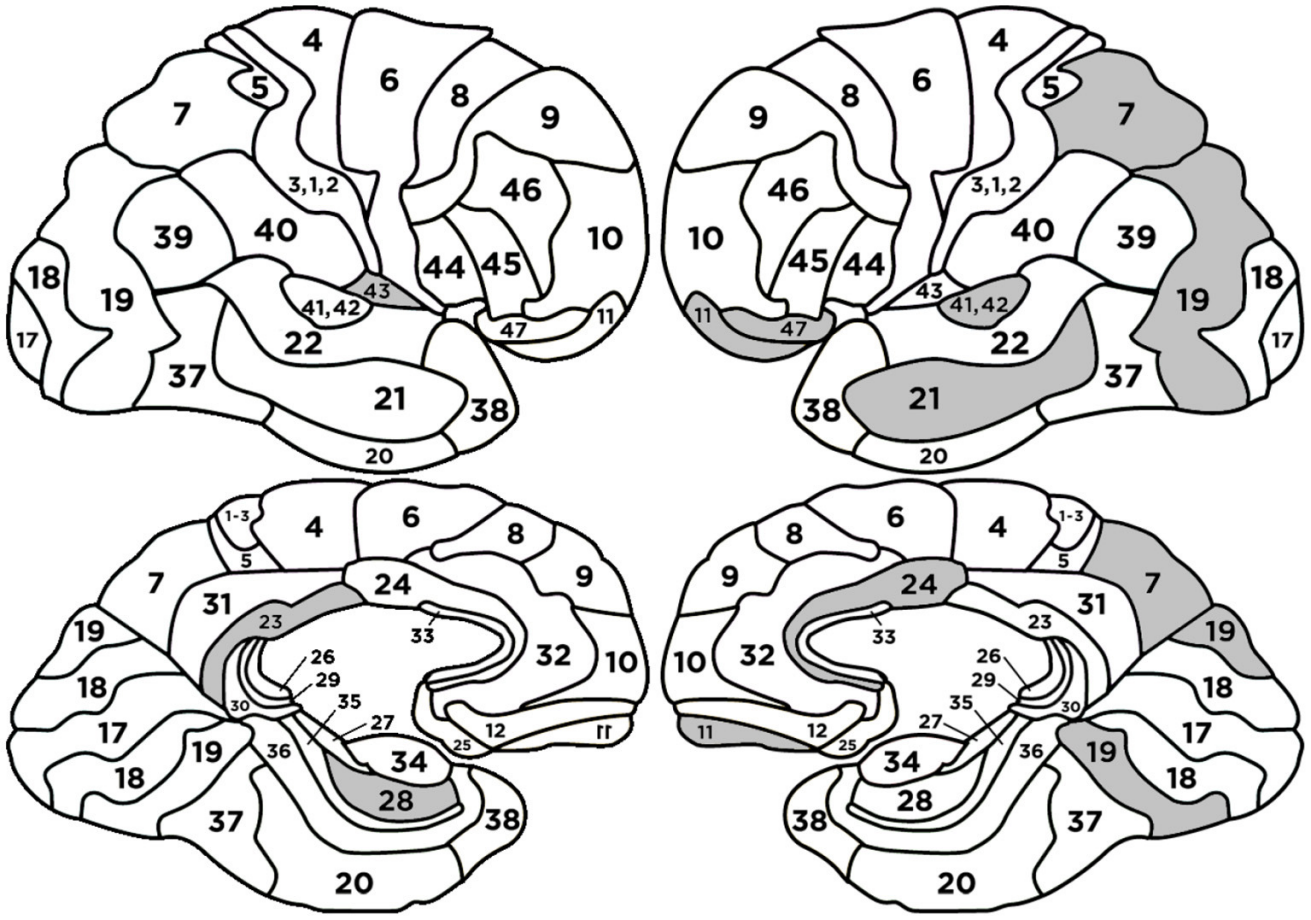
Heads with marked BAs that have the largest impact on the classification of source credibility evaluations (cases SC and SNC).

The following BAs: L-BA07, R-BA42, L-BA47, L-BA24, L-BA21 had positive regression parameters β equal to 1.631, 1.240, 1.237, 1.058, 0.679, respectively. The following BAs: L-BA11, R-BA43, L-BA19, R-BA23, R-BA28 had negative regression parameters β equal to –1.435, –1.396, –1.139, –0.758, and –0.606, respectively. The anatomical structures and their functionalities are presented in Appendix 4 and highlighted in Figure 7.

The presented results prove that we can build a good model for predicting source credibility, based on results of stage 2 of our experiment.

**Hypothesis 5 verification**

To verify hypothesis 5, we compared the models prepared to verify hypotheses 3 and 4.

Qualitative indicators were compared for each model (see Table 6).

**Table 6:** Comparing quality measures of logistic regression models for predicting message credibility evaluations and source credibility evaluations.

	**The best model for predicting message credibility evaluations**	**The best model for predicting source credibility evaluations**
Accuracy	0.74	0.71
Precision	0.75	0.72
Recall	0.71	0.70
F1	0.73	0.71

The BAs that have the largest impact on message credibility evaluations (used in models for hypothesis 3) and on source credibility evaluations (used in models for hypothesis 4) are presented in Table 7.

**Table 7:** Comparing BAs that had the largest impact on the classification of MC and MNC in the best model for predicting message credibility evaluations with the BAs that had the largest impact on the classification of SC and SNC in the best model for predicting source credibility evaluations.

**Hypothesis 3**	**Hypothesis 4**
L-BA24	L-BA07
L-BA28	L-BA11
L-BA35	L-BA19
L-BA39	L-BA21
R-BA36	L-BA24
L-Hippocampus	L-BA47
	R-BA23
	R-BA28
	R-BA42
	R-BA43

*The BAs that are the same for both models are marked in green. The BAs that have the same number for both models but different hemispheres of the brain (L or R) are marked in orange*.

The results presented in Tables 6, 7 show that there are differences between the models for hypotheses 3 and 4, which proves hypothesis 5.

**Hypothesis 6 verification**

In Kawiak et al. (2020b), we found ROIs that have impact on decision making based on source credibility. We decided to map the ROI to BAs to check whether the detected BAs from another experiment could be successfully used as input data for the model built in hypothesis 4. As a result, from 10 ROI we obtained 36 BAs (see Appendix Section 3.1). We compared the results of the model from Kawiak et al. (2020b) with the results from hypothesis 4 (see Table 8).

**Table 8:** Comparing quality measures of logistic regression models from Kawiak et al. (2020b) and hypothesis 4.

**Models**	**Nb of BA**	**ACC**	**Precision**	**Recall**	**F1**
ROI	36	0.70	0.71	0.70	0.70
Hypothesis 4	10	0.71	0.72	0.70	0.71

**Figure 8:**
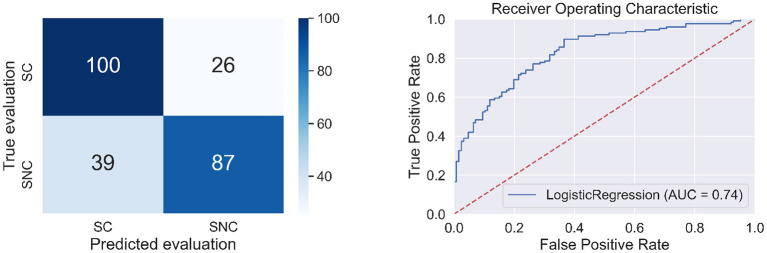
Confusion matrix (left side) and the ROC curve (right side) for the model from hypothesis 6.

Then we built a model for the classification of source credibility evaluations (based on cases: $SC=C_{S1}^{P2}\cup C_{S3}^{P2}$ and $SNC=NC_{S1}^{P2}\cup NC_{S3}^{P2}$) using the BAs from Kawiak et al. (2020b) as input data. The result of the classifier is presented in Table 9 and Figure 8.

**Table 9:** Quality measures of the logistic regression model for predicting source credibility evaluations using BAs from Kawiak et al. (2020b) as independent variables.

Accuracy	0.74
Precision	0.77
Recall	0.69
F1	0.73

The above results prove that we can build a good model for predicting source credibility evaluations using BAs from Kawiak et al. (2020b) as independent variables.

## 5. Discussion

The results of our experiment confirmed our expectation that it is possible to distinguish and predict credibility evaluations based on brain activity registered using EEG. The Mann-Whitney statistical test showed that there are statistically significant differences between MEC in all BAs. This finding is in agreement with previous work. Dimoka noticed that different BAs in the brain are activated when we trust and distrust a person (Dimoka, 2010). Previous work on source credibility (Kawiak et al., 2020a,b) also reported similar findings.

Our experiment has been composed of two parts. In the first part, participants made credibility evaluations based mainly on message content. For the cases that involved student S2 who had an average accuracy of 50%, participants had no relevant information about source credibility. In the second part, participants could take into account their learned information about source credibility of the three students (S1, S2, and S3). Especially for S1 (average accuracy of 25%) and S3 (average accuracy of 75%), participants made their credibility evaluations based on source credibility. Our observations confirmed the role of source credibility in the second part of the experiment, which confirms the internal validity of the experiment.

In the first stage of the experiment, participants needed to evaluate credibility based on message content. This process required, among other things, language processing, memory encoding and retrieval, and calculation. These activities are reflected in the activity of BAs when the classification of MC and MNC is made by the machine learning algorithm. The BAs: L-BA24, L-BA39, L-BA35, R-BA36, L-Hippocampus, L-BA28 had the largest impact on the classification of MC and MNC (see Appendix 4). The L-BA24 is responsible, among others, for language processing. This BA is well described in the literature (Ardila et al., 2016). During the experiment, the participant's brains processed information. Data was encoded in memory and retrieved back. The following BAs are responsible for the encoding process: L-BA28, L-BA35, R-BA36, L-Hippocampus (Kircher et al., 2007; Yan et al., 2021) and for the retrieval process the L-BA35 field. Rajah and McIntosh (2005) and Maguire et al. (2000). The L-BA35 field contributes to both the encoding and information retrieval process (Dougal et al., 2007). The L-BA39 is responsible for mathematical calculation (Grabner et al., 2007). The activity of this BA probably resulted from the fact that participants tried to count good and wrong answers of the students to gain knowledge about students' accuracy in answering questions.

In the second stage of the experiment, participants dealt with Japanese signs, translating signs, making decisions, recalling information about the student. These activities are reflected in the activity of BAs when the classification of SC and SNC is made by the machine learning algorithm. The BAs: L-BA07, R-BA42, R-BA47, L-BA24, L-BA21, L-BA11, R-BA43, L-BA19, R-BA23, R-BA28 had the largest impact on the classification of SC and SNC (see Appendix 4). The presence of the R-BA42 field, which is largely responsible for the auditory processing, was a big surprise (Appendix 4). This BA is also responsible for repetition priming (Haist et al., 2001). The activity of this BA probably results from the fact that the respondents formed an opinion about a given student through repeated correct or incorrect answers. For the classification of SC and SNC, the R-BA23 appears. It is responsible for the evaluative judgment (Zysset et al., 2002). The participants of our experiment had to decide whether they trusted the answers of student S1, S2, or S3. They must assess the source credibility. The BAs: L-BA47, R-BA43, L-BA07, L-BA24, L-BA19, L-BA21 are responsible, among others, for language processing. These BAs are well described in the literature (Ardila et al., 2016). The L-BA19 is responsible for processing Japanese signs. Söderfeldt et al. (1997), Horwitz et al. (2003), and Ardila et al. (2015).

Comparing the BAs with the largest impact on the classification of MC and MNC in the best model for predicting message credibility evaluations with the BAs that had the largest impact on the classification of SC and SNC in the best model for predicting source credibility evaluations, it can be noticed that L-BA24 appears in both models. The presence of the same BA in both models shows that there are common elements between the two stages of the experiment. These elements include language expression and working memory.

In this article, we compared the results of the classifier to predicting source credibility decisions with the classifier from Kawiak et al. (2020b). Analyzing the classifier's independent variables, it can be noticed that some BAs are common. These BAs are: L-BA07, L-BA11, L-BA24, L-BA47, R-BA23, R-BA42, R-BA43. The presence of a large number of common BAs proves the correctness of the stage of experiment concerning the source credibility and the existence of common elements in both experiments.

The L-BA07 (Superior parietal gyrus) is located in the parietal lobe and except of its widely reported somato-sensory functions may be involved in language processing, even as reported in Li et al. (2015) Chonese character processing which may refer to our Japanese signs in experiment.

Brodmann Areas L-BA11, L-BA24 and L-BA47 are in prefrontal cortex and are involved in executive tasks, attention, and memory. They may be involved in the postulated in Kawiak et al. (2020b) credibility loop as the credibility evaluation, especially the source credibility, is associated more or less with previous experience, recalling from memory, etc.

However, in the group of BAa mentioned above the most interesting seem to be the R-BA23 (Posterior cingulate cortex), R-BA42 (Secondary auditory cortex) and R-BA43 (Postcentral gyrus). They are all located in the right hemisphere relatively close to each other.

The R-BA23 according to Technologies (2012) associated with evaluative judgement is also reported in Beer et al. (2008) where they model Implicit Association Test (IAT) to examine the automatic processes that contribute to social attitudes including prejudice in their fMRI studies. Remember that after the first stage of experiment participants could get prejudiced to weak or average student.

The R-BA42 according to Technologies (2012) is associated among others with working memory, visual speech perception and several auditory acoustic and speech related patterns. However, also in the fMRI studies it has been shown by Arsalidou et al. (2013) that R-BA42 activity varies in function of task difficulty engaging working memory. It is to be investigated whether and when the credibility evaluation is difficult enough to activate/deactivate R-BA42. Nevertheless, we suppose it may be one of the most important in the credibility loop.

The R-BA43 according to Technologies (2012) is associated with spoken language bilaterally. It is also found to play a role in distributed face recognition system network (Cloutier et al., 2017) and in female groups investigated in fMRI studies (Toffoletto et al., 2014) where emotional and cognitive functional imaging of estrogen and progesterone effects were discussed and for the R-BA43 they were reported in Goldstein et al. (2005). In that fMRI studies the difference in BOLD signal intensity between aversive affective to neutral visual stimuli during early follicular vs. midcycle menstrual phases in R-BA43 was noted.

So we hypothesize that gathering together working memory in processing prejudices, faces, task difficulties and aversive, affective and neutral stimuli may result in association of the L-BA07, R-BA23, R-BA42 and R-BA43 also with credibility evaluation. However, this requires intensive fMRI studies.

## 6. Conclusion and Future Work

Our results confirm that using the source localization algorithms (sLORETA) and machine learning classifiers it is possible to predict message credibility evaluation with and without the perfect knowledge, and construct a model involving finite number of variables to achieve accuracy around 0.7. However, the research described herein still can be considered as an initial stage of larger series of experiments leading to the precise indication of the so-called credibility loop postulated by us in Kawiak et al. (2020b) to function in the brain. The main limitation is still a relatively small number of participants that makes it possible to gather full recording of their EEG activity during the whole experiment. On the other hand, our results confirm some similarities found in our other independent experiments reported, i.e., in Kawiak et al. (2020b) which may indicate that we are proceeding in good direction.

In the next step we will investigate a larger group of participants in order to gain possibility of building universal models able to predict decisions taken by people from outside the cohort. Our first attempts have been promising, and evaluating our approach for the signal collected from new and completely separated from the training and validation sub-cohorts subjects for tests give the average accuracy of 0.65 for both stages of the experiment, and it will be reported in future.

It will be useful in future to engage convolutional neural networks and deep learning methods that can improve the efficiency of standard AI classifiers by several percent.

In future research it may be worth consideration to totally move to the regions of interest (ROI) mappings instead of using Brodmann Areas (BA). The remapping one to another is not trivial, however, moving to ROI may make it possible to find similarities in other researchers findings.

Finally, when the credibility loop is more precisely estimated we hope to probe it more deeply using functional MRI scanning.

## Data Availability Statement

The raw data supporting the conclusions of this article will be made available by the authors, without undue reservation.

## Ethics Statement

The studies involving human participants were reviewed and approved by University's Bioethical Commission (MCSU Bioethical Commission permission 13.06.2019. The patients/participants provided their written informed consent to participate in this study.

## Author Contributions

PS: idea of the experiment, classifiers construction, modeling, and simulation. GW: experiment idea, writing manuscripts, modeling idea, and MEC idea. All authors contributed to the manuscript, data analysis and worked in the laboratory equally.

## Conflict of Interest

The authors declare that the research was conducted in the absence of any commercial or financial relationships that could be construed as a potential conflict of interest.

## Publisher's Note

All claims expressed in this article are solely those of the authors and do not necessarily represent those of their affiliated organizations, or those of the publisher, the editors and the reviewers. Any product that may be evaluated in this article, or claim that may be made by its manufacturer, is not guaranteed or endorsed by the publisher.

## Footnotes

^1^ https://toolbox.google.com/factcheck/explorer

^2^ https://blog.twitter.com/en_us/topics/product/2021/introducing-birdwatch-a-community-based-approach-to-misinformation

^3^Electrical Geodesic Systems, Inc., 500 East 4th Ave. Suite 200, Eugene, OR 97401, USA.

## Appendix

### A. Additions for Hypothesis 3

#### A.1. List of 32 Selected BAs After Using RFE With CV for Hypothesis 3

R-BA06, L-BA07, L-BA10, L-BA11, R-BA11, L-BA17, L-BA18, R-BA19, L-BA22, L-BA24, L-BA25, L-BA28, L-BA30, L-BA32, R-BA32, L-BA33, L-BA35, R-BA36, L-BA37, R-BA37, R-BA38, L-BA39, L-BA41, R-BA41, R-BA42, L-BA43, L-BA44, R-BA44, L-BA45, R-BA45, L-BA47, L-Hippocampus.

### B. Additions for Hypothesis 4

#### B.1. List of 26 Selected BAs After Using RFE With CV for Hypothesis 4

L-BA07, L-BA11, L-BA19, R-BA20, L-BA21, R-BA22, L-BA23, R-BA23, L-BA24, R-BA24, L-BA25, R-BA28, L-BA29, R-BA30, L-BA35, R-BA36, L-BA37, L-BA38, R-BA38, L-BA41, R-BA42, L-BA43, R-BA43, R-BA44, L-BA46, L-BA47.

### C. Additions for Hypothesis 6

#### C.1. List of 36 BAs Mapped From ROI

L-BA02, R-BA02, L-BA04, R-BA04, L-BA06, R-BA06, L-BA07, R-BA07, L-BA10, R-BA10, L-BA11, R-BA11, L-BA13, R-BA13, L-BA23, R-BA23, L-BA24, R-BA24, L-BA31, R-BA31, L-BA32, R-BA32, L-BA33, R-BA33, L-BA39, R-BA39, L-BA41, R-BA41, L-BA42, R-BA42, L-BA43, R-BA43, L-BA44, R-BA44, L-BA47, RBA47.

### D. List of BAs With Selected Features

The full list of BAs and their cortical functions can be found in [41].

**Table:**

**BA**	**Anatomical structure**	**Known functions**
L-BA07	Somatosensory association cortex	Working memory, Conscious recollection of previously experienced events, language processing, processing emotions and self-reflections during decision making
L-BA11	Orbitofrontal area	Decision making involving reward
L-BA19	Associative visual cortex (V3)	Detection of patterns, word and face encoding, sign language
L-BA21	Middle temporal gyrus	Sentence generation, word generation, deductive reasoning
R-BA23	Posterior cingulate gyrus	Evaluative judgment
L-BA24	Anterior cingulate gyrus	Language expression, working memory
L-BA28	Ventral entorhinal cortex	Memory encoding, working memory
R-BA28	Ventral entorhinal cortex	Memory encoding, working memory
L-BA35	Perirhinal cortex	Memory encoding and retrieval
R-BA36	Hippocampal area	Memory encoding, working memory
L-BA39	Angular gyrus	Calculation
R-BA42	Primary and auditory association cortex	Repetition priming effect, auditory working memory, visual speech perception, processing discontinued acoustic patterns
R-BA43	Primary gustatory cortex	Spoken language (Bilateral)
L-BA47	Inferior prefontal gyrus	Semantic processing, semantic encoding, active semantic retrieval, single-word reading, working memory, deductive reasoning
L-Hippocampus	Hippocampus	Regulating learning, memory encoding, memory consolidation
